# (Meth­oxy­methyl­idene)di­methyl­aza­nium tetra­phenyl­borate aceto­nitrile monosolvate

**DOI:** 10.1107/S1600536814003481

**Published:** 2014-02-22

**Authors:** Ioannis Tiritiris, Stefan Saur, Willi Kantlehner

**Affiliations:** a Fakultät Chemie/Organische Chemie, Hochschule Aalen, Beethovenstrasse 1, D-73430 Aalen, Germany

## Abstract

In the cation of the title salt, C_4_H_10_NO^+^·C_24_H_20_B^−^·C_2_H_3_N, the C—N bond lengths are 1.2864 (16), 1.4651 (17) and 1.4686 (16) Å, indicating double- and single-bond character, respectively. The C—O bond length of 1.2978 (15) Å shows double-bond character, pointing towards charge delocalization within the NCO plane of the iminium ion. C—H⋯π inter­actions are present between the methine H atom and two of the phenyl rings of the tetra­phenyl­borate ion. The latter forms an aromatic pocket in which the cation is embedded. The iminium ion is further connected through a C—H⋯N hydrogen bond to the aceto­nitrile mol­ecule. This leads to the formation of a two-dimensional supramolecular pattern along the *bc* plane.

## Related literature   

For the crystal structures of alkali metal tetra­phenyl­borates, see: Behrens *et al.* (2012 ▶). For the synthesis of 1,3-dioxolanes and 1,3-dioxanes from meth­oxy­methyl­ene-*N*,*N*- di­methyl­iminium methyl sulfate, diols and carbonyl compounds, see: Kantlehner & Gutbrod (1979 ▶). For the synthesis of acetals from meth­oxy­methyl­ene-*N*,*N*-di­methyl­iminium methyl sulfate, alcohols and aliphatic or aromatic aldehydes, see: Kantlehner *et al.* (1974 ▶).
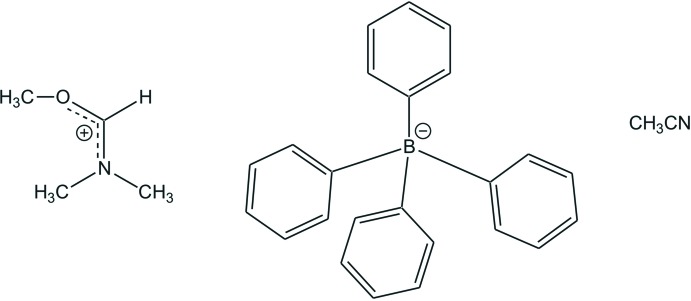


## Experimental   

### 

#### Crystal data  

C_4_H_10_NO^+^·C_24_H_20_B^−^·C_2_H_3_N
*M* *_r_* = 448.39
Monoclinic, 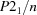

*a* = 10.6715 (5) Å
*b* = 16.9824 (9) Å
*c* = 14.4061 (7) Å
β = 103.515 (3)°
*V* = 2538.5 (2) Å^3^
*Z* = 4
Mo *K*α radiation
μ = 0.07 mm^−1^
*T* = 100 K
0.20 × 0.15 × 0.10 mm

#### Data collection  

Bruker Kappa APEXII DUO diffractometer
54637 measured reflections
7810 independent reflections
5762 reflections with *I* > 2σ(*I*)
*R*_int_ = 0.045

#### Refinement  

*R*[*F* ^2^ > 2σ(*F* ^2^)] = 0.050
*wR*(*F* ^2^) = 0.136
*S* = 1.01
7810 reflections
315 parameters
H atoms treated by a mixture of independent and constrained refinement
Δρ_max_ = 0.42 e Å^−3^
Δρ_min_ = −0.43 e Å^−3^

### 

Data collection: *APEX2* (Bruker, 2008 ▶); cell refinement: *SAINT* (Bruker, 2008 ▶); data reduction: *SAINT*; program(s) used to solve structure: *SHELXS97* (Sheldrick, 2008 ▶); program(s) used to refine structure: *SHELXL97* (Sheldrick, 2008 ▶); molecular graphics: *DIAMOND* (Brandenburg & Putz, 2005 ▶); software used to prepare material for publication: *SHELXL97*.

## Supplementary Material

Crystal structure: contains datablock(s) I, global. DOI: 10.1107/S1600536814003481/zl2579sup1.cif

Structure factors: contains datablock(s) I. DOI: 10.1107/S1600536814003481/zl2579Isup2.hkl

CCDC reference:

Additional supporting information:  crystallographic information; 3D view; checkCIF report

## Figures and Tables

**Table 1:** Hydrogen-bond geometry (Å, °) *Cg*1 and *Cg*2 are the centroids of the C17–C22 and C23–C28 rings, respectively.

*D*—H⋯*A*	*D*—H	H⋯*A*	*D*⋯*A*	*D*—H⋯*A*
C3—H3⋯*Cg*1	0.94 (2)	2.75 (2)	3.542 (2)	143 (2)
C3—H3⋯*Cg*2	0.94 (2)	2.88 (2)	3.272 (2)	106 (2)
C2—H2*B*⋯N2	0.98	2.66	3.640 (2)	178

**Fig. 1.**
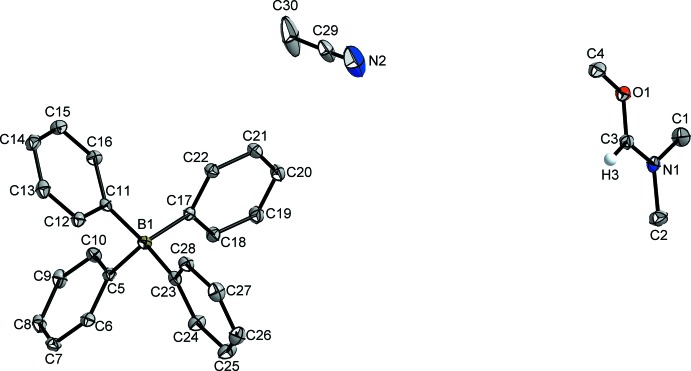
The structure of the title compound with displacement ellipsoids at the 50% probability level. All carbon bonded hydrogen (except of H3) atoms were omitted for the sake of clarity.

**Fig. 2.**
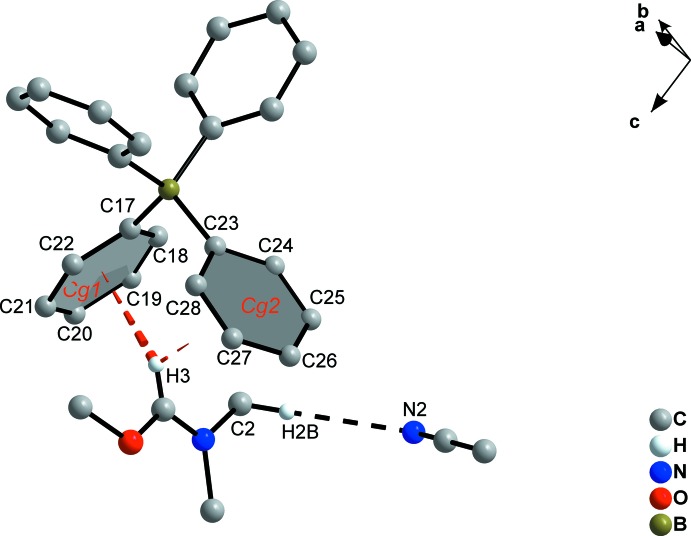
C–H···π interactions (red dashed lines) between the hydrogen atom H3 of the cation and the phenyl carbon atoms (centroids) of the tetraphenylborate ion and C–H···N hydrogen bond (black dashed line) between the cation and the acetonitrile molecule.

## supplementary crystallographic information

### 1. Comment

The ionic liquid methoxymethylene-*N*,*N*-dimethyliminium methyl sulfate is a 1:1 adduct of *N*,*N*-dimethylformamide and dimethyl sulfate. It reacts with mixtures of alcohols and aliphatic or aromatic aldehydes giving acetals (Kantlehner *et al.*, 1974). It reacts also with mixtures of carbonyl compounds and 1,2- as well as 1,3-dioles, giving 1,3-dioxolanes and 1,3-dioxanes respectively (Kantlehner *et al.*, 1979). By reacting methoxymethylene-*N*,*N*-dimethyliminium methyl sulfate with sodium tetraphenylborate, it was possible to achieve an anion exchange and to obtain the title compound. According to the structure analysis, the C1–N1 bond length is 1.4686 (16) Å, C2–N1 = 1.4651 (17) Å and C3–N1 = 1.2864 (16) Å, showing single and double bond character, respectively. The C–N1–C angles are: 117.79 (10)° (C1–N1–C2), 121.54 (11)° (C1–N1–C3) and 120.64 (11)° (C3–N1–C2), which indicates a nearly trigonal-planar surrounding of the nitrogen centre by the carbon atoms (Fig. 1). The C–O bond length shows with 1.2978 (15) Å double bond character. The positive charge is completely delocalized within the plane formed by the atoms N1, C3 and O1. The bond lengths and angles in the tetraphenylborate ion are in good agreement with the data from the crystal structure analysis of the alkali metal tetraphenylborates (Behrens *et al.*, 2012). C–H···π interactions between the hydrogen atom H3 of the cation and two phenyl rings (centroids) of the tetraphenylborate ion are present (Fig. 2), with hydrogen centroid distances of 2.75 and 2.88 Å (Tab. 1). The phenyl rings form aromatic pockets, in which the iminium ion is embedded. The cation is further connected through a C–H···N hydrogen bond (Fig. 2) with the acetonitrile molecule [*d*(H2B···N2) = 2.66 Å] (Tab. 1). This leads to the formation of a two-dimensional supramolecular pattern along the *bc* plane.

### 2. Experimental

The title compound was obtained by reacting equimolar amounts of *N*,*N*-dimethylformamide with dimethyl sulfate at room temperature forming methoxymethylene-*N*,*N*-dimethyliminium methyl sulfate (I). 1.00 g (5.01 mmol) of crude (I) was dissolved in 20 ml acetonitrile and 1.72 g (5.01 mmol) of sodium tetraphenylborate in 20 ml acetonitrile was added. After stirring for one hour at room temperature, the precipitated sodium methyl sulfate was filtered off. The title compound crystallized from a saturated acetonitrile solution after several days at 273 K, forming colorless single crystals suitable for X-ray analysis.

Dimethyl sulfate is carcinogenic, mutagenic and highly poisonous. During use appropriate precautions should be taken.

### 3. Refinement

The H atom bound to C3 was located in a difference Fourier map and was refined freely [C—H = 0.94 (2) Å]. The hydrogen atoms of the methyl groups were allowed to rotate with a fixed angle around the C–N and C–O bonds to best fit the experimental electron density, with *U*_iso_(H) set to 1.5*U*_eq_(C) and d(C—H) = 0.98 Å. The H atoms in the aromatic rings were placed in calculated positions with (C—H) = 0.95 Å. They were included in the refinement in the riding model approximation, with *U*_iso_(H) set to 1.2 *U*_eq_(C).

### Crystal data

**Table:**

C_4_H_10_NO^+^·C_24_H_20_B^−^·C_2_H_3_N	*F*(000) = 960
*M**_r_* = 448.39	*D*_x_ = 1.173 Mg m^−^^3^
Monoclinic, *P*2_1_/*n*	Mo *K*α radiation, λ = 0.71073 Å
Hall symbol: -P 2yn	Cell parameters from 54637 reflections
*a* = 10.6715 (5) Å	θ = 1.9–30.6°
*b* = 16.9824 (9) Å	µ = 0.07 mm^−^^1^
*c* = 14.4061 (7) Å	*T* = 100 K
β = 103.515 (3)°	Block, colorless
*V* = 2538.5 (2) Å^3^	0.20 × 0.15 × 0.10 mm
*Z* = 4	

### Data collection

**Table:**

Bruker Kappa APEXII DUO diffractometer	5762 reflections with *I* > 2σ(*I*)
Radiation source: fine-focus sealed tube	*R*_int_ = 0.045
Graphite monochromator	θ_max_ = 30.6°, θ_min_ = 1.9°
φ scans, and ω scans	*h* = −15→15
54637 measured reflections	*k* = −24→24
7810 independent reflections	*l* = −20→20

### Refinement

**Table:**

Refinement on *F*^2^	Primary atom site location: structure-invariant direct methods
Least-squares matrix: full	Secondary atom site location: difference Fourier map
*R*[*F*^2^ > 2σ(*F*^2^)] = 0.050	Hydrogen site location: inferred from neighbouring sites
*wR*(*F*^2^) = 0.136	H atoms treated by a mixture of independent and constrained refinement
*S* = 1.01	*w* = 1/[σ^2^(*F*_o_^2^) + (0.0606*P*)^2^ + 1.2901*P*] where *P* = (*F*_o_^2^ + 2*F*_c_^2^)/3
7810 reflections	(Δ/σ)_max_ < 0.001
315 parameters	Δρ_max_ = 0.42 e Å^−^^3^
0 restraints	Δρ_min_ = −0.43 e Å^−^^3^

### Special details

**Table:**

Geometry. All e.s.d.'s (except the e.s.d. in the dihedral angle between two l.s. planes) are estimated using the full covariance matrix. The cell e.s.d.'s are taken into account individually in the estimation of e.s.d.'s in distances, angles and torsion angles; correlations between e.s.d.'s in cell parameters are only used when they are defined by crystal symmetry. An approximate (isotropic) treatment of cell e.s.d.'s is used for estimating e.s.d.'s involving l.s. planes.
Refinement. Refinement of *F*^2^ against ALL reflections. The weighted *R*-factor *wR* and goodness of fit *S* are based on *F*^2^, conventional *R*-factors *R* are based on *F*, with *F* set to zero for negative *F*^2^. The threshold expression of *F*^2^ > σ(*F*^2^) is used only for calculating *R*-factors(gt) *etc*. and is not relevant to the choice of reflections for refinement. *R*-factors based on *F*^2^ are statistically about twice as large as those based on *F*, and *R*- factors based on ALL data will be even larger.

### Fractional atomic coordinates and isotropic or equivalent isotropic displacement parameters (Å^2^)

**Table:**

	*x*	*y*	*z*	*U*_iso_*/*U*_eq_	
C1	0.46641 (13)	0.40926 (8)	0.41370 (10)	0.0232 (3)	
H1A	0.4462	0.3829	0.4689	0.035*	
H1B	0.3895	0.4364	0.3773	0.035*	
H1C	0.5355	0.4476	0.4357	0.035*	
C2	0.40869 (13)	0.30622 (9)	0.28573 (11)	0.0250 (3)	
H2A	0.4494	0.2710	0.2476	0.037*	
H2B	0.3514	0.3429	0.2434	0.037*	
H2C	0.3586	0.2751	0.3216	0.037*	
N1	0.50842 (10)	0.35056 (6)	0.35249 (8)	0.0164 (2)	
C3	0.62856 (12)	0.33664 (8)	0.35858 (9)	0.0170 (2)	
H3	0.6545 (15)	0.2972 (10)	0.3213 (12)	0.022 (4)*	
O1	0.71519 (8)	0.37622 (6)	0.41880 (6)	0.0192 (2)	
C4	0.84859 (12)	0.35136 (9)	0.43104 (10)	0.0243 (3)	
H4A	0.8737	0.3187	0.4885	0.036*	
H4B	0.9044	0.3978	0.4377	0.036*	
H4C	0.8575	0.3208	0.3752	0.036*	
B1	0.76218 (12)	0.20090 (8)	0.12815 (9)	0.0131 (2)	
C5	0.69730 (11)	0.15845 (7)	0.02587 (8)	0.0132 (2)	
C6	0.66415 (11)	0.20044 (7)	−0.06029 (9)	0.0153 (2)	
H6A	0.6699	0.2563	−0.0582	0.018*	
C7	0.62318 (11)	0.16379 (8)	−0.14890 (9)	0.0175 (2)	
H7A	0.6021	0.1946	−0.2055	0.021*	
C8	0.61317 (12)	0.08245 (8)	−0.15432 (9)	0.0197 (3)	
H8A	0.5849	0.0570	−0.2143	0.024*	
C9	0.64509 (12)	0.03865 (8)	−0.07057 (9)	0.0190 (3)	
H9A	0.6388	−0.0171	−0.0733	0.023*	
C10	0.68623 (12)	0.07615 (7)	0.01730 (9)	0.0162 (2)	
H10A	0.7076	0.0449	0.0735	0.019*	
C11	0.91812 (11)	0.19238 (7)	0.13791 (8)	0.0136 (2)	
C12	0.98740 (12)	0.24623 (7)	0.09522 (9)	0.0168 (2)	
H12A	0.9434	0.2908	0.0635	0.020*	
C13	1.11786 (12)	0.23712 (8)	0.09739 (9)	0.0196 (3)	
H13A	1.1610	0.2756	0.0684	0.024*	
C14	1.18458 (12)	0.17234 (8)	0.14155 (10)	0.0210 (3)	
H14A	1.2737	0.1660	0.1437	0.025*	
C15	1.11920 (13)	0.11662 (8)	0.18281 (10)	0.0212 (3)	
H15A	1.1634	0.0713	0.2125	0.025*	
C16	0.98875 (12)	0.12694 (8)	0.18076 (9)	0.0178 (2)	
H16A	0.9461	0.0881	0.2095	0.021*	
C17	0.71395 (11)	0.16101 (7)	0.21767 (8)	0.0141 (2)	
C18	0.58786 (12)	0.13210 (8)	0.20676 (9)	0.0171 (2)	
H18A	0.5346	0.1279	0.1441	0.021*	
C19	0.53758 (13)	0.10938 (8)	0.28364 (10)	0.0209 (3)	
H19A	0.4517	0.0903	0.2728	0.025*	
C20	0.61303 (14)	0.11463 (9)	0.37614 (10)	0.0237 (3)	
H20A	0.5791	0.1001	0.4291	0.028*	
C21	0.73893 (14)	0.14146 (9)	0.38990 (9)	0.0229 (3)	
H21A	0.7920	0.1447	0.4527	0.027*	
C22	0.78792 (12)	0.16375 (8)	0.31212 (9)	0.0177 (2)	
H22A	0.8746	0.1815	0.3234	0.021*	
C23	0.71577 (11)	0.29311 (7)	0.13220 (8)	0.0139 (2)	
C24	0.58709 (12)	0.31596 (8)	0.09488 (10)	0.0194 (3)	
H24A	0.5284	0.2782	0.0606	0.023*	
C25	0.54234 (13)	0.39139 (8)	0.10609 (10)	0.0241 (3)	
H25A	0.4547	0.4042	0.0794	0.029*	
C26	0.62499 (14)	0.44803 (8)	0.15603 (10)	0.0230 (3)	
H26A	0.5948	0.4997	0.1638	0.028*	
C27	0.75235 (13)	0.42791 (8)	0.19436 (10)	0.0207 (3)	
H27A	0.8102	0.4659	0.2290	0.025*	
C28	0.79607 (12)	0.35215 (7)	0.18238 (9)	0.0166 (2)	
H28A	0.8839	0.3399	0.2093	0.020*	
N2	0.2002 (2)	0.44404 (10)	0.12761 (13)	0.0599 (5)	
C29	0.21400 (16)	0.50210 (9)	0.09583 (10)	0.0284 (3)	
C30	0.2352 (4)	0.57636 (13)	0.0566 (2)	0.0899 (11)	
H30A	0.2142	0.6185	0.0969	0.135*	
H30B	0.1801	0.5811	−0.0079	0.135*	
H30C	0.3258	0.5807	0.0538	0.135*	

### Atomic displacement parameters (Å^2^)

**Table:**

	*U*^11^	*U*^22^	*U*^33^	*U*^12^	*U*^13^	*U*^23^
C1	0.0197 (6)	0.0234 (7)	0.0280 (7)	0.0057 (5)	0.0084 (5)	−0.0057 (5)
C2	0.0168 (6)	0.0288 (7)	0.0291 (7)	−0.0024 (5)	0.0049 (5)	−0.0071 (6)
N1	0.0154 (5)	0.0165 (5)	0.0182 (5)	0.0012 (4)	0.0058 (4)	0.0003 (4)
C3	0.0163 (5)	0.0193 (6)	0.0163 (6)	0.0007 (5)	0.0054 (4)	−0.0001 (5)
O1	0.0145 (4)	0.0243 (5)	0.0190 (4)	0.0004 (3)	0.0043 (3)	−0.0037 (4)
C4	0.0133 (6)	0.0354 (8)	0.0236 (7)	0.0010 (5)	0.0035 (5)	−0.0061 (6)
B1	0.0135 (6)	0.0123 (6)	0.0128 (6)	−0.0008 (4)	0.0019 (5)	−0.0006 (5)
C5	0.0109 (5)	0.0146 (5)	0.0141 (5)	−0.0006 (4)	0.0031 (4)	−0.0004 (4)
C6	0.0141 (5)	0.0161 (6)	0.0154 (5)	−0.0011 (4)	0.0029 (4)	0.0010 (4)
C7	0.0127 (5)	0.0261 (7)	0.0132 (5)	−0.0013 (5)	0.0017 (4)	0.0022 (5)
C8	0.0156 (5)	0.0278 (7)	0.0157 (6)	−0.0037 (5)	0.0036 (4)	−0.0073 (5)
C9	0.0199 (6)	0.0168 (6)	0.0209 (6)	−0.0028 (5)	0.0061 (5)	−0.0056 (5)
C10	0.0176 (5)	0.0156 (6)	0.0155 (6)	−0.0012 (4)	0.0039 (4)	0.0000 (4)
C11	0.0143 (5)	0.0146 (5)	0.0112 (5)	−0.0003 (4)	0.0015 (4)	−0.0023 (4)
C12	0.0172 (5)	0.0166 (6)	0.0162 (6)	0.0006 (4)	0.0035 (4)	0.0003 (4)
C13	0.0183 (6)	0.0216 (6)	0.0201 (6)	−0.0041 (5)	0.0069 (5)	−0.0021 (5)
C14	0.0134 (5)	0.0286 (7)	0.0206 (6)	0.0010 (5)	0.0034 (5)	−0.0039 (5)
C15	0.0193 (6)	0.0238 (7)	0.0195 (6)	0.0065 (5)	0.0023 (5)	0.0019 (5)
C16	0.0178 (6)	0.0179 (6)	0.0179 (6)	0.0016 (5)	0.0043 (5)	0.0011 (5)
C17	0.0170 (5)	0.0119 (5)	0.0134 (5)	0.0018 (4)	0.0035 (4)	−0.0010 (4)
C18	0.0174 (5)	0.0183 (6)	0.0156 (6)	−0.0006 (5)	0.0037 (4)	−0.0001 (4)
C19	0.0208 (6)	0.0223 (6)	0.0217 (6)	0.0006 (5)	0.0092 (5)	0.0019 (5)
C20	0.0292 (7)	0.0262 (7)	0.0189 (6)	0.0061 (6)	0.0123 (5)	0.0047 (5)
C21	0.0277 (7)	0.0272 (7)	0.0131 (6)	0.0070 (5)	0.0036 (5)	0.0018 (5)
C22	0.0177 (5)	0.0193 (6)	0.0151 (6)	0.0025 (5)	0.0019 (4)	−0.0010 (5)
C23	0.0152 (5)	0.0148 (5)	0.0119 (5)	−0.0013 (4)	0.0034 (4)	−0.0007 (4)
C24	0.0156 (5)	0.0180 (6)	0.0227 (6)	−0.0003 (5)	0.0007 (5)	−0.0053 (5)
C25	0.0197 (6)	0.0217 (7)	0.0284 (7)	0.0052 (5)	0.0004 (5)	−0.0056 (5)
C26	0.0282 (7)	0.0154 (6)	0.0254 (7)	0.0025 (5)	0.0064 (5)	−0.0039 (5)
C27	0.0249 (6)	0.0157 (6)	0.0214 (6)	−0.0049 (5)	0.0053 (5)	−0.0049 (5)
C28	0.0165 (5)	0.0170 (6)	0.0156 (6)	−0.0020 (4)	0.0022 (4)	−0.0020 (4)
N2	0.1152 (17)	0.0315 (9)	0.0441 (10)	−0.0065 (9)	0.0412 (11)	−0.0027 (7)
C29	0.0433 (9)	0.0261 (7)	0.0184 (6)	0.0057 (6)	0.0126 (6)	0.0001 (5)
C30	0.191 (3)	0.0343 (11)	0.0705 (17)	0.0081 (16)	0.084 (2)	0.0175 (11)

### Geometric parameters (Å, º)

**Table:**

C1—N1	1.4686 (16)	C13—C14	1.3822 (19)
C1—H1A	0.9800	C13—H13A	0.9500
C1—H1B	0.9800	C14—C15	1.3889 (19)
C1—H1C	0.9800	C14—H14A	0.9500
C2—N1	1.4651 (17)	C15—C16	1.3966 (17)
C2—H2A	0.9800	C15—H15A	0.9500
C2—H2B	0.9800	C16—H16A	0.9500
C2—H2C	0.9800	C17—C22	1.4053 (17)
N1—C3	1.2864 (16)	C17—C18	1.4062 (17)
C3—O1	1.2978 (15)	C18—C19	1.3933 (17)
C3—H3	0.940 (17)	C18—H18A	0.9500
O1—C4	1.4555 (15)	C19—C20	1.389 (2)
C4—H4A	0.9800	C19—H19A	0.9500
C4—H4B	0.9800	C20—C21	1.388 (2)
C4—H4C	0.9800	C20—H20A	0.9500
B1—C5	1.6411 (17)	C21—C22	1.3949 (18)
B1—C17	1.6423 (18)	C21—H21A	0.9500
B1—C11	1.6433 (17)	C22—H22A	0.9500
B1—C23	1.6477 (18)	C23—C28	1.4044 (17)
C5—C6	1.4033 (17)	C23—C24	1.4072 (17)
C5—C10	1.4056 (17)	C24—C25	1.3897 (18)
C6—C7	1.3954 (17)	C24—H24A	0.9500
C6—H6A	0.9500	C25—C26	1.3872 (19)
C7—C8	1.3862 (19)	C25—H25A	0.9500
C7—H7A	0.9500	C26—C27	1.3850 (19)
C8—C9	1.3903 (19)	C26—H26A	0.9500
C8—H8A	0.9500	C27—C28	1.3930 (18)
C9—C10	1.3933 (17)	C27—H27A	0.9500
C9—H9A	0.9500	C28—H28A	0.9500
C10—H10A	0.9500	N2—C29	1.111 (2)
C11—C16	1.4023 (17)	C29—C30	1.421 (3)
C11—C12	1.4049 (17)	C30—H30A	0.9800
C12—C13	1.3938 (17)	C30—H30B	0.9800
C12—H12A	0.9500	C30—H30C	0.9800
			
N1—C1—H1A	109.5	C14—C13—C12	120.20 (12)
N1—C1—H1B	109.5	C14—C13—H13A	119.9
H1A—C1—H1B	109.5	C12—C13—H13A	119.9
N1—C1—H1C	109.5	C13—C14—C15	118.88 (12)
H1A—C1—H1C	109.5	C13—C14—H14A	120.6
H1B—C1—H1C	109.5	C15—C14—H14A	120.6
N1—C2—H2A	109.5	C14—C15—C16	120.22 (12)
N1—C2—H2B	109.5	C14—C15—H15A	119.9
H2A—C2—H2B	109.5	C16—C15—H15A	119.9
N1—C2—H2C	109.5	C15—C16—C11	122.64 (12)
H2A—C2—H2C	109.5	C15—C16—H16A	118.7
H2B—C2—H2C	109.5	C11—C16—H16A	118.7
C3—N1—C2	120.64 (11)	C22—C17—C18	115.22 (11)
C3—N1—C1	121.54 (11)	C22—C17—B1	122.58 (11)
C2—N1—C1	117.79 (10)	C18—C17—B1	121.57 (10)
N1—C3—O1	119.55 (12)	C19—C18—C17	122.96 (12)
N1—C3—H3	120.9 (10)	C19—C18—H18A	118.5
O1—C3—H3	119.5 (10)	C17—C18—H18A	118.5
C3—O1—C4	117.03 (10)	C20—C19—C18	120.01 (12)
O1—C4—H4A	109.5	C20—C19—H19A	120.0
O1—C4—H4B	109.5	C18—C19—H19A	120.0
H4A—C4—H4B	109.5	C21—C20—C19	118.85 (12)
O1—C4—H4C	109.5	C21—C20—H20A	120.6
H4A—C4—H4C	109.5	C19—C20—H20A	120.6
H4B—C4—H4C	109.5	C20—C21—C22	120.42 (12)
C5—B1—C17	112.33 (10)	C20—C21—H21A	119.8
C5—B1—C11	104.15 (9)	C22—C21—H21A	119.8
C17—B1—C11	113.17 (10)	C21—C22—C17	122.52 (12)
C5—B1—C23	112.46 (10)	C21—C22—H22A	118.7
C17—B1—C23	102.26 (9)	C17—C22—H22A	118.7
C11—B1—C23	112.79 (9)	C28—C23—C24	115.05 (11)
C6—C5—C10	115.41 (11)	C28—C23—B1	122.99 (10)
C6—C5—B1	122.49 (10)	C24—C23—B1	121.53 (10)
C10—C5—B1	121.65 (10)	C25—C24—C23	122.75 (12)
C7—C6—C5	122.88 (12)	C25—C24—H24A	118.6
C7—C6—H6A	118.6	C23—C24—H24A	118.6
C5—C6—H6A	118.6	C26—C25—C24	120.35 (12)
C8—C7—C6	119.97 (12)	C26—C25—H25A	119.8
C8—C7—H7A	120.0	C24—C25—H25A	119.8
C6—C7—H7A	120.0	C27—C26—C25	118.78 (12)
C7—C8—C9	118.97 (12)	C27—C26—H26A	120.6
C7—C8—H8A	120.5	C25—C26—H26A	120.6
C9—C8—H8A	120.5	C26—C27—C28	120.29 (12)
C8—C9—C10	120.34 (12)	C26—C27—H27A	119.9
C8—C9—H9A	119.8	C28—C27—H27A	119.9
C10—C9—H9A	119.8	C27—C28—C23	122.78 (12)
C9—C10—C5	122.44 (12)	C27—C28—H28A	118.6
C9—C10—H10A	118.8	C23—C28—H28A	118.6
C5—C10—H10A	118.8	N2—C29—C30	178.5 (3)
C16—C11—C12	115.14 (11)	C29—C30—H30A	109.5
C16—C11—B1	122.47 (11)	C29—C30—H30B	109.5
C12—C11—B1	122.09 (10)	H30A—C30—H30B	109.5
C13—C12—C11	122.89 (12)	C29—C30—H30C	109.5
C13—C12—H12A	118.6	H30A—C30—H30C	109.5
C11—C12—H12A	118.6	H30B—C30—H30C	109.5
			
C2—N1—C3—O1	−179.44 (12)	B1—C11—C16—C15	175.04 (12)
C1—N1—C3—O1	−1.32 (19)	C5—B1—C17—C22	154.41 (11)
N1—C3—O1—C4	172.58 (12)	C11—B1—C17—C22	36.81 (15)
C17—B1—C5—C6	145.07 (11)	C23—B1—C17—C22	−84.80 (13)
C11—B1—C5—C6	−92.10 (12)	C5—B1—C17—C18	−35.14 (15)
C23—B1—C5—C6	30.35 (15)	C11—B1—C17—C18	−152.73 (11)
C17—B1—C5—C10	−43.00 (15)	C23—B1—C17—C18	85.66 (13)
C11—B1—C5—C10	79.83 (13)	C22—C17—C18—C19	1.48 (18)
C23—B1—C5—C10	−157.72 (10)	B1—C17—C18—C19	−169.64 (12)
C10—C5—C6—C7	−0.15 (17)	C17—C18—C19—C20	−0.1 (2)
B1—C5—C6—C7	172.26 (11)	C18—C19—C20—C21	−1.1 (2)
C5—C6—C7—C8	0.34 (18)	C19—C20—C21—C22	0.9 (2)
C6—C7—C8—C9	−0.31 (18)	C20—C21—C22—C17	0.6 (2)
C7—C8—C9—C10	0.12 (19)	C18—C17—C22—C21	−1.68 (18)
C8—C9—C10—C5	0.07 (19)	B1—C17—C22—C21	169.34 (12)
C6—C5—C10—C9	−0.06 (17)	C5—B1—C23—C28	−146.49 (11)
B1—C5—C10—C9	−172.53 (11)	C17—B1—C23—C28	92.80 (13)
C5—B1—C11—C16	−89.05 (13)	C11—B1—C23—C28	−29.06 (16)
C17—B1—C11—C16	33.23 (15)	C5—B1—C23—C24	41.38 (15)
C23—B1—C11—C16	148.72 (11)	C17—B1—C23—C24	−79.33 (13)
C5—B1—C11—C12	84.26 (13)	C11—B1—C23—C24	158.81 (11)
C17—B1—C11—C12	−153.46 (11)	C28—C23—C24—C25	0.38 (19)
C23—B1—C11—C12	−37.98 (15)	B1—C23—C24—C25	173.10 (12)
C16—C11—C12—C13	−1.88 (18)	C23—C24—C25—C26	−0.3 (2)
B1—C11—C12—C13	−175.65 (11)	C24—C25—C26—C27	−0.1 (2)
C11—C12—C13—C14	1.05 (19)	C25—C26—C27—C28	0.2 (2)
C12—C13—C14—C15	0.47 (19)	C26—C27—C28—C23	−0.1 (2)
C13—C14—C15—C16	−1.0 (2)	C24—C23—C28—C27	−0.20 (18)
C14—C15—C16—C11	0.1 (2)	B1—C23—C28—C27	−172.80 (12)
C12—C11—C16—C15	1.30 (18)		

### Hydrogen-bond geometry (Å, º)

Cg1 and Cg2 are the centroids of the C17–C22 and C23–C28 rings, respectively.

**Table:**

*D*—H···*A*	*D*—H	H···*A*	*D*···*A*	*D*—H···*A*
C3—H3···*Cg*1	0.94 (2)	2.75 (2)	3.542 (2)	143 (2)
C3—H3···*Cg*2	0.94 (2)	2.88 (2)	3.272 (2)	106 (2)
C2—H2*B*···N2	0.98	2.66	3.640 (2)	178
